# Glucose Oxidase/Egg White Protein Microparticles with a Redox Mediator for Glucose Biosensors on a Screen-Printed Electrode and a Decomposable Electrode

**DOI:** 10.3390/bios13080772

**Published:** 2023-07-29

**Authors:** Natcha Rasitanon, Parinthorn Rattanapan, Kanyawee Kaewpradub, Chittanon Buranachai, Itthipon Jeerapan

**Affiliations:** 1 Center of Excellence for Trace Analysis and Biosensor, Prince of Songkla University, Hat Yai 90110, Thailand; 6510220094@email.psu.ac.th (N.R.); parinthorn.ra@st.wu.ac.th (P.R.); 6510220131@email.psu.ac.th (K.K.); chittanon.b@psu.ac.th (C.B.); 2 Division of Physical Science, Faculty of Science, Prince of Songkla University, Hat Yai 90110, Thailand; 3 Center of Excellence for Innovation in Chemistry, Faculty of Science, Prince of Songkla University, Hat Yai 90110, Thailand

**Keywords:** glucose oxidase, egg white proteins, biosensors, glucose, 9,10-phenanthrenequinone, particles, degradable electrodes

## Abstract

Glucose oxidase (GOx) is a typical model enzyme used to create biosensors. Exploring a strategy to prepare ready-to-use functional enzymatic microparticles combining GOx and food-based proteins offers compelling advantages. However, no reports exist on the integration of egg white materials to synthesize functional biorecognition particles with glucose oxidation catalytic functions for electrochemical biosensors. Here, we demonstrate functional microparticles combining egg white proteins, GOx, and 9,10-phenanthrenequinone (PQ). The egg white proteins crosslink to form three-dimensional scaffolds to accommodate GOx and redox molecules. The PQ mediator enhances electron transfer between the electrode surface and the GOx enzyme’s flavin adenine dinucleotides. The functional microparticles are directly applied to the printed electrode. The performance of these microparticles is evaluated using a screen-printed carbon nanotube (CNT)-modified electrode coated with GOx/PQ/egg white protein microparticles. The analytical performance of the system exhibits a linear range of 0.125−40 mM, with a maximum current ($I_{max})$ and a Michaelis–Menten constant $\left(K_{m}\right)$ being 0.2 µA and 4.6 mM, respectively. Additionally, a decomposable electrode composed of CNTs and edible oil conjugated with functional enzyme microparticles is shown to undergo degradation under gastric conditions. Utilizing food-based proteins to accommodate enzymes and to create redox-active microparticles for catalyzing glucose oxidation offers advantages in developing affordable and degradable bioelectrodes. This concept holds promise for advancing biocompatible electrodes in biosensor and bioelectronics applications.

## 1. Introduction

Food-based materials have become increasingly popular in biomedical applications, sensors, and environmentally friendly “green” applications. This is primarily due to their biocompatibility, biodegradability, and low toxicity [1]. An example of this “green” strategy involved utilizing edible materials, such as carbon composites as conductors and olive oil as binders, to create disposable electrochemical sensors [2]. These sensors are capable of measuring uric acid, ascorbic acid, and dopamine, which are commonly found in saliva, gastric fluids, or intestinal fluids. Another example is the use of biodegradable electrodes that incorporate interconnected three-dimensional conductive nanocomposites (e.g., single-wall carbon nanotubes (SWCNTs)), graphene, and conductive polymers within edible starch–chitosan substrates [3]. The starch and chitosan components were sourced from potatoes and crab shells. These electrodes, based on the starch–chitosan substrate, exhibited rapid biodegradation in a lysozyme solution at room temperature, leaving no toxic residues behind. Additionally, another composite paste relying on biodegradable conductive oleogel was demonstrated [4]. The materials were composed of food-grade beeswax, vegetable oil, and activated carbon conductive fillers. Biodegradable conductive oleogel composites showed antibacterial and hydrophobic properties, making them suitable for contact with food and ensuring the prevention of contamination while providing food’s sensor capabilities. The promising performance of recently developed sensors, composed of food-based materials, suggests significant potential for advancing environmentally friendly electronics or diagnostic tools designed for biochemical monitoring purposes.

In addition to utilizing food-based materials, it is essential to explore strategies for immobilizing enzymes in affordable materials. This exploration is crucial for enabling practical biosensing applications because an important process to create a biosensor is enzyme immobilization, which involves attaching enzymes to solid supports through physical adsorption, entrapment within a matrix or encapsulation, or covalent bonding [5]. The immobilization of enzymes offers several advantages, including the repeatable usage of biomolecules and improving their stability [6]. The delicate nature of enzymes poses a significant challenge in producing functional enzyme-based materials. Enzymes are inherently fragile and prone to losing their catalytic activity if mishandled or exposed to extreme environments. Various materials, including lignin [7], chitosan [8], and egg white proteins [9], have been utilized to enhance enzyme immobilization and to improve their stability. The immobilized enzymes demonstrated higher activity when compared with free enzymes. In addition to stability, it is necessary to devise a strategy for synthesizing readily available functional enzymatic materials that can be utilized as a biocatalytic layer on desired electrodes. The development of such a method simplifies fabrication processes, saves time, and enhances accessibility for customization according to specific requirements. Consequently, considerable efforts have been directed towards the development of effective strategies for immobilizing enzymes using solid supports made from biomaterials.

Biomaterial microparticles (MPs) are of great interest in various applications, including encapsulation, tissue engineering, and biosensing, due to their advantageous structural, environmental protection, and functional abilities as a carrier system [10,11]. While the majority of MPs are made from synthetic polymers (such as poly(glycolic acid), poly(lactic acid), poly(p-dioxanone), and copolymers of trimethylene carbonate and glycolide) [12], exploring the utilization of inexpensive biomaterials is crucial in expanding the possibilities for developing biocompatible MPs with functional properties. An example of mesoporous silica (SiO_2_) MPs derived from CNTs was synthesized using a one-pot spray pyrolysis process, providing efficient support for the enzyme immobilization [13]. The resulting porous SiO_2_ MPs, functionalized with glutaraldehyde, were utilized for immobilizing lipase. This immobilization process led to improved biochemical properties (higher activity and stability at a wide range of pH values, and harsh organic solvent concentrations) and enhanced thermostability of the lipase. The mesoporous SiO_2_-bound lipase was used to construct a lipase biosensor for triglyceride. However, this work does not integrate food-based materials as degradable electrodes, and the method of synthesis described in this approach requires a high temperature, as high as 500 °C. Importantly, naturally derived materials such as cellulose, chitosan, and silk are suitable for “green” strategies. One example relied on the use of cellulose and chitosan conjugated with magnetic microspheres, which were prepared using the sol–gel transition method, with ionic liquids serving as the solvent for cellulose and chitosan dissolution and regeneration [14]. These composite microspheres were also activated with glutaraldehyde to immobilize glucose oxidase (GOx). The resulting GOx-immobilized microspheres exhibited excellent catalytic activity for glucose oxidation. However, their potential applications in electrochemical sensors were not investigated. Another example is the use of silk coatings on poly(lactic-co-glycolic acid) and alginate microspheres for encapsulating horseradish peroxidase and tetramethylrhodamine-conjugated bovine serum albumin (BSA) [15]. Another strategy involves the emulsification–internal gelation process, which can immobilize GOx onto the surface of alginate microspheres [16]. Free carboxyl groups present in alginate microspheres activate carbodiimide and N-hydroxysuccinimide, enabling the covalent immobilization of GOx. This process leads to the strong binding of the GOx to the alginate microsphere’s surface through the formation of amide bonds. Although this study described the synthesis of sodium alginate microspheres, the size distribution of particles varied. No efforts have been made yet to develop electrochemical glucose sensor applications.

In addition to those approaches, the application of a calcium carbonate (CaCO_3_) template for preparing protein MPs has been reported. For example, one fabrication method used coprecipitation to entrap protein molecules such as BSA inside spherical CaCO_3_ templates [17]. This concept relied on the intracellular disulfide interchange reactions at physiological conditions mediated by glutathione to initiate disulfide interchange reactions between the BSA molecules. After the glutathione and CaCO_3_ templates were removed, the entrapped BSA molecules form new intermolecular disulfide bonds with neighboring molecules, resulting in BSA MPs. Afterward, BSA-horseradish peroxidase MPs were prepared to show the colorimetric reaction between horseradish peroxidase and 3,3’,5,5’-tetramethylbenzidine in the presence of H_2_O_2_ (an oxidizing agent). This existing report focused on colorimetric and fluorescence techniques, excluding electrochemical applications. In addition to the use of BSA, the use of hen eggs has previously been reported to synthesize hollow MPs [18]. It can be fabricated using high-density lipoproteins in egg yolk and sacrificial CaCO_3_ templates. This work utilized a redox reaction between Au^3+^ ions and proteins, which resulted in the formation of a protein gel network. However, to the best of our knowledge, no efforts have been made to demonstrate the use of food-based particles with enzymatic functions for degradable and electrochemical biosensors. Therefore, further research is required to explore food-based materials with enzymatic particles and to advance the technology towards environmentally friendly synthesis, inexpensive production, and practical implementation in biodegradable electronics.

**Figure 1:**
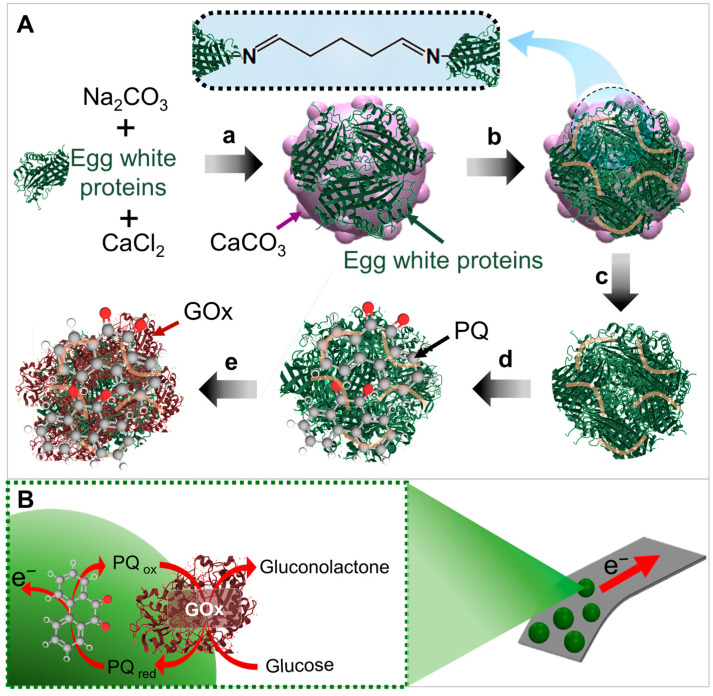
Schematic diagram of glucose biosensors based on GOx/egg white protein MPs with a redox mediator. (**A**) Illustration of synthesis of a GOx/PQ/egg white protein MP: (**a**) Loading egg white proteins in a CaCO_3_ template. (**b**) Crosslinking egg white protein MPs. (**c**) Removing the CaCO_3_ template. (**d**) Functionalizing a PQ mediator. (**e**) Immobilizing GOx to obtain a PQ/egg white protein MP. (**B**) The components of a screen-printed electrode functionalized with GOx/PQ/egg white protein MPs along with the redox reactions occurring on its surface.

This work presents the first demonstration of GOx/(9,10-phenanthrenequinone, PQ)/egg white protein MPs, which can be used to develop glucose sensors (Figure 1). The egg white protein MPs were crosslinked with glutaraldehyde to generate a 3D scaffold structure, which increases the active site availability for GOx as the enzyme immobilization and enhances the analytical performance. Note that egg white proteins are inexpensive, costing 0.003 USD per 1 g, in contrast to the price of other common proteins, such as BSA (25 USD per 1 g). In this work, the egg white protein MPs were bound with PQ as a mediator, facilitating electron transfer between an electrode and the GOx enzyme. Additionally, the PQ/egg white protein MPs were immobilized with GOx to develop glucose sensors. We demonstrated a proof-of-concept model of GOx coupled with food-based materials in the form of MPs. This is because a glucose sensor plays a crucial role in monitoring glucose levels, especially in the context of diabetes management. It provides individuals with valuable information to make informed decisions regarding their diet, medication, and overall well-being [19]. Impedance and amperometry techniques were utilized to demonstrate the capability of glucose-sensitive MPs (i.e., GOx/PQ/egg white protein MPs). Additionally, the electrochemical properties of degradable electrodes fabricated using carbon materials mixed with edible olive oil and coated with GOx/PQ/egg white protein MPs were investigated toward glucose detection. These types of MP and electrode exhibit the potential for degradation. Moreover, these sensors prove to be cost-effective and environmentally friendly biomaterials. These advancements open up promising future applications in various fields, such as healthcare, biotechnology, and personal wellness.

## 2. Materials and Methods

### 2.1. Chemicals and Materials

Multiwalled CNTs (95% purity, diameter = 5–15 nm, length = 10–30 µm) were from Luoyang Advanced Material Co., Ltd., Shanghai, China. D−(+)-glucose anhydrous was from Fluka, Neu-Ulm, Germany. GOx (from *Aspergillus niger*, type VII, 248,878 units g^−1^), L-ascorbic acid, L-lactic acid, uric acid, creatinine, chitosan (from shrimp shell, ≥75%), potassium hexacyanoferrate (III) and glutaraldehyde solution (grade II, 25% in H_2_O) were from Sigma-Aldrich (Saint Louis, MO, USA). Potassium phosphate dibasic (K_2_HPO_4_) and potassium phosphate monobasic (KH_2_PO_4_) were from BDH Laboratory Supplies Poole, UK. Sodium carbonate (Na_2_CO_3_) and calcium chloride dihydrate (CaCl_2_·2H_2_O) were from Ajax Finechem (Seven Hills, New South Wales, Australia). Toluene was from Guangdong Guanghua Chemical Factory Co., Ltd., Haizhu District, Guangdong, China. Ethanol was from RCl Labscan Ltd. (Bangkok, Thailand). Acetone was from VWR International Ltd. (Poole, UK). Chicken eggs (large size, weight 55–65 g) were brought from Sangthong Saha Farm Company Limited, Thailand. PQ was from Sigma Aldrich, Darmstadt, Germany. 2-propanol and acetic acid were from Avantor Performance Material, Inc., Taiwan. Butan-1-ol was from RCI Labscan Limited, Bangkok, Thailand. Olive oil was brought from CARAPELLI FIRENZE S.p.A. Via L.Da Vinci, Tavarnelle Val Di Pesa Firenze, Italy. Potassium hexacyanoferrate (II) trihydrate was brought from RDH Laborchemikalien GmbH & Co. KG., Hamburg, Germany.

All chemical solutions were prepared using deionized water (18.2 MΩ cm) from a Milli Q Merck system (Darmstadt, Germany). A 0.1 M potassium phosphate-buffered solution (PBS) with a pH of 7.0 was prepared using KH_2_PO_4_ and K_2_HPO_4_, and it was employed as the supporting electrolyte. All electrochemical performance was studied in a 0.1 M PBS solution with a pH of 7.0, unless stated otherwise. The artificial gastric fluid preparation was achieved by using 1 g of NaCl with 4 mL of HCl and by adjusting the volume to 500 mL using water [20].

### 2.2. Instruments and Electrochemical Measurement

A µStat-I 400 (Metrohm Dropsens, S.L., Asturias, Spain) controlled via DropView8400 software version 3.78 was used to examine electrochemical performances, including cyclic voltammetry (CV) and amperometry. Electrochemical impedance spectroscopy (EIS) was performed using μAutolab Type III FRA 2, Metrohm controlled via NOVA software version 1.11. The EIS characterizations were conducted at glucose concentrations of 0, 0.5, 5, and 10 mM at a potential of 0.2 V, employing a frequency range of 10^0^–10^4^ Hz with 50 sample points in between in a logarithmic scale and an amplitude of 0.005 V. Working electrodes (such as screen-printing electrodes coated with GOx/PQ/egg white protein MPs) were evaluated with Pt counter and Ag/AgCl (3.0 M KCl) reference electrodes during the three-electrode-system analysis. The amperometry response was measured, after 30 s of immersion in the test solution, at constant potential at 0.2 V (vs. Ag/AgCl) for 30 s. Calibration plots were obtained by increasing the glucose concentration in 0.1 M PBS, pH 7.0. Infrared spectra (IR) were measured using an FTS165 FTIR spectrometer. Fluorescence measurements were conducted using an F-2700 fluorometer from Hitachi High-Tech Science Corporation, Tokyo, Japan. Optical microscopy images were captured using an Olympus IX70 microscope from Japan.

### 2.3. Preparation of GOx/PQ/Egg White Protein MPs

To prepare the desired functional biocatalytic MPs, porous, biocompatible, and decomposable CaCO_3_ MPs were co-synthesized with egg white proteins. This initial step aimed to encapsulate the egg white proteins, which would later be transformed into a biofriendly three-dimensional (3D) scaffold capable of accommodating active components. The CaCO_3_ MPs served as a hard microtemplate to ensure a uniform shape for the synthesized functional particles, as illustrated in Figure 1A. First, 1 mL of egg white proteins was mixed with 1.5 mL of 1 M Na_2_CO_3_ solution and rapidly stirred at 600 rpm for 2 min using a magnetic stirrer, while 1.5 mL of 1 M CaCl_2_ solution was added into the mixture (Figure 1(Aa)). The egg white proteins were extracted directly and slowly using a 24-gauge syringe needle with a length of 38 mm from a fresh egg. The resulting white precipitate (CaCO_3_ MPs loaded with egg white proteins) was subsequently washed with 2-propanol, ethanol, and water. After each washing step, the cleaned CaCO_3_ MPs loaded with egg white proteins were collected via centrifugation at 3000 rpm for 1 min at room temperature. Subsequently, the CaCO_3_ MPs loaded with egg white proteins were washed three times with 400 µL of 1.5 M NaCl and centrifuged at 3000 rpm for 5 min at room temperature (25 °C). Next, the CaCO_3_ templates loaded with egg white proteins were incubated with 1 mL of 0.5% *v*/*v* glutaraldehyde for 6 h using a tube plate rotator (Figure 1(Ab)). After that, precipitates were washed with water through centrifugation at 3000 rpm for 1 min at room temperature (25 °C). Following this step, the CaCO_3_ template was removed by adjusting the pH of the suspended egg white protein MPs/CaCO_3_ with the addition 400 µL of 0.2 M HCl solution (Figure 1(Ac)). The resulting egg white protein MPs were collected via centrifugation at 3000 rpm for 1 min at room temperature and washed with water and butan-1-ol. Then, 400 μL of butan-1-ol and vortex was added. After that, egg white protein MPs were centrifuged at 3000 rpm for 5 min at room temperature (25 °C) to collect the precipitate of crosslinked egg white protein MPs. To introduce a redox mediator into the egg white protein MPs, 750 μL of 5 mM PQ was added to incubate it with egg white protein MPs for 2 h and using a tube plate rotator (Figure 1(Ad)). The excess PQ was removed through centrifugation at 3000 rpm for 1 min, and the resulting mixture was redispersed with butan-1-ol for washing. After that, the supernatant was removed, and the PQ/egg white protein MP precipitants were collected. Subsequently, the PQ-egg white protein MPs were incubated with 750 µL of 0.5% *v*/*v* glutaraldehyde for 30 min using a tube plate rotator and removing the excess glutaraldehyde through centrifugation at 3000 rpm for 1 min. Finally, GOx solution (10 mg mL^−1^ GOx dissolved in 10% *v*/*v* of egg white in 0.05 M PBS, pH 7.0) was then added to the PQ/egg white protein MPs and incubated for 2 h to immobilize the enzyme in the MPs using a tube plate rotator (Figure 1(Ae)). The excess of GOx was removed through centrifugation at 3000 rpm for 1 min and redispersed with 750 µL of 0.05 mM PBS pH 7.0. In this process, GOx/PQ/egg white protein MPs were obtained, which were dispersed in 0.05 M PBS at pH 7.0. These MPs were ready to use for electrode modification.

### 2.4. Preparation of CNT-Modified Carbon Ink

To prepare a CNT-modified ink, 60 mg of CNTs was dispersed in 490 µL of toluene using a homogenizer probe (model AR-0975) level 1 for 3 min. Next, 7 g of the carbon conductive ink (Guangzhou Print Area Technology Co. Ltd., Guangzhou, China) was mixed with the mixture using a mixing machine (Shashin Kagaku, Kyoto, Japan, Kakuhunter, SK-300SII) for 30 min at 2000 rpm. The resulting CNT-modified carbon ink was printed on a polyethylene terephthalate sheet as a working electrode. The working area of the working electrode was 0.3 cm × 0.5 cm. The electrode was electrochemically cleaned by applying a constant voltage of 1.50 V for 60 s in 1.0 M Na_2_CO_3_.

### 2.5. Preparation of Biosensors on Degradable Electrodes

The use of olive oil in constructing a carbon paste electrode has been reported [2]. In this work, the degradable electrodes for glucose determination were prepared using CNT as the conductive filler material and edible olive oil as a binder. To prepare a degradable electrode, 30 mg of CNT and 30 μL of olive oil were thoroughly hand-mixed using a mortar and pestle. A portion of the resulting mixed paste was then packed into a tube with a diameter of 0.4 cm and a length of 0.5 cm. For electrochemical measurements, electrical contacts were established by inserting a conductive stainless-steel wire into the top side of the packed paste. To functionalize the biosensor, 10 µL of the GOx/PQ/egg white protein MPs was dropped on the modified electrode and dried. After that, 10 µL of 1.5% *wt/v* chitosan in 0.25 M acetic acid was dropped onto the electrode and dried at room temperature.

## 3. Results and Discussion

### 3.1. Principle of Preparation GOx/PQ/Egg White Protein MPs

Encapsulating egg white proteins with a CaCO_3_ template involved coprecipitation, where CaCO_3_ MPs were formed simultaneously with egg white proteins. In this strategy, egg white proteins were initially dissolved in an aqueous solution containing calcium ions (Ca^2+^). Following that, carbonate ions (CO_3_^2−^) were added to bind with Ca^2+^ and to form solid CaCO_3_ particles. These solid particles acted as a trap, encapsulating the egg white protein molecules within them (Figure 1(Aa)). After the egg white proteins were entrapped inside the CaCO_3_ particles, the loaded particles were crosslinked using glutaraldehyde (Figure 1(Ab)). This process involved treating proteins, mainly ovalbumin, with glutaraldehyde, which formed strong covalent bonds between the amino groups of the protein and the aldehyde groups of glutaraldehyde, resulting in a cross-linked network. The protein in egg white underwent a conformational transition, changing from a water-soluble form to a water-insoluble form. The crosslinked ovalbumin could serve as a scaffold for the subsequent immobilization of enzymes. Subsequently, the sacrificial CaCO_3_ MPs were dissolved using an acidic solution, resulting in the formation of egg white protein MPs that maintained the spherical shape of the original CaCO_3_ particles (Figure 1(Ac)). During this step, the CaCO_3_ particles broke down, and Ca^2+^ ions were released along with carbon dioxide. This process is widely employed in various applications, including material synthesis, because an acid solution can selectively dissolve and remove CaCO_3_ without affecting other materials or surfaces. It is an inexpensive and easy-to-implement method [21].

To showcase an application of the food-based protein MPs, a glucose biosensor was presented as an example. The subsequent functionalization of essential components, including redox molecules and GOx, into the egg white protein MPs, was demonstrated.

After achieving crosslinked egg white protein MPs, the particles were incubated with PQ (Figure 1(Ad)). PQ is an organic compound that serves as a redox mediator, facilitating electron transfer between the electrode surface and the enzyme. It consists of a phenanthrene ring fused with a quinone moiety [22]. Quinones belong to a class of organic compounds characterized by a conjugated cyclic system with two carbonyl groups [23,24]. They can undergo reversible reductions, both one-electron and two-electron-based, resulting in the generation of a semiquinone radical or a quinone, respectively [25]. The mediated function of a quinone compound plays a critical role in enhancing the efficiency and sensitivity of biosensing applications. It facilitates the transfer of electrons involved in electrochemical reactions. Moreover, a mediator-based glucose biosensor improves electron transfer, reduces interference effects, and mitigates the impact of oxygen compared with the first generation of enzymatic glucose biosensors [26]. Therefore, our sensor utilized the second generation of enzymatic glucose sensors with PQ as the mediator.

To create glucose-sensitive MPs, we immobilized PQ/egg white protein MPs with GOx, an enzyme that catalyzes the oxidation of glucose (Figure 1(Ae)). This immobilization technique improved the stability of the enzyme, making it well-suited for glucose biosensors. GOx is composed of two identical 80 kDa subunits and is associated with two non-covalently bound flavin adenine dinucleotides. The flavin adenine dinucleotides coenzyme serves as an electron carrier and facilitates the oxidation of D-glucose. This reaction yields D-gluconolactone and H_2_O_2_ as products, while the mediator PQ facilitates electron transfer between the enzyme and the electrode surface (Figure 1B). Once the PQ/GOx/egg white protein MPs were obtained, they were ready to be applied in glucose biosensor applications. 

### 3.2. Characterization and Morphology

**Figure 2:**
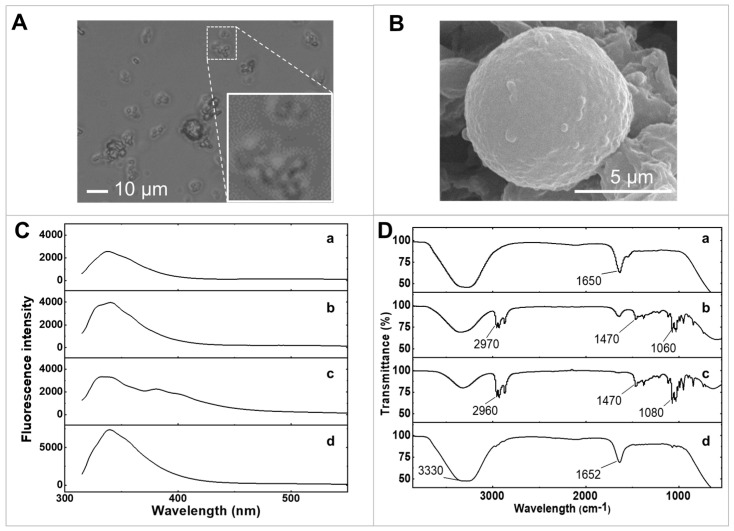
Characterization of materials. (**A**) The optical microscopic image of GOx/PQ/egg white protein MPs. (**B**) SEM image of a screen-printed electrode coated with GOx/PQ/egg white protein MPs. (**C**) Fluorescence emission spectrum of (**a**) egg white proteins, (**b**) egg white protein MPs, (**c**) PQ/egg white protein MPs, and (**d**) GOx/PQ/egg white protein MPs at emission wavelengths from 315 to 550 nm. (**D**) FTIR spectra of (**a**) egg white proteins, (**b**) egg white protein MPs, (**c**) PQ/egg white protein MPs, and (**d**) GOx/PQ/egg white protein MPs.

Optical microscopy was employed to examine the morphologies of GOx/PQ/egg white protein MPs once the CaCO_3_ template was eliminated. This method enabled visual observation and analysis of the physical structures and shapes of the MPs. Figure 2A shows that egg white protein MPs exhibit a uniform spherical shape with a narrow size distribution, characterized by an average diameter of 5 µm. Additionally, the corresponding CaCO_3_ MPs loaded with egg white proteins before template removal demonstrate a similar size distribution, with an average diameter of 5 µm (Figure S1).

The morphologies of egg white protein MPs loaded with PQ and GOx were examined using scanning electron microscopy (SEM) (Figure 2B). The GOx/PQ/egg white protein MPs exhibited a spherical structure. During the coprecipitation process involving CaCl_2_ and Na_2_CO_3_, nucleation led to the formation of granules, which subsequently assembled into interconnected spherical structures with pores. This arrangement facilitated the encapsulation of many molecules of egg white proteins within the template. The uniform shape and small size of these spherical MPs offer advantages such as a high surface-to-volume ratio, enabling enhanced contact with target substances and electrode surfaces. Additionally, their homogeneity ensures consistent performance.

By studying changes in the fluorescence emission spectrum with excitation wavelength at 300 nm, it is possible to examine molecular interactions such as binding to a target molecule or undergoing conformational changes (Figure 2C). We investigated the fluorescence emission spectra of raw egg white, egg white protein MPs, PQ/egg white protein MPs, and GOx/PQ/egg white protein MPs, under an emission wavelength range of 315–550 nm. The fluorescence emission spectra reveal that crosslinking with glutaraldehyde did not significantly alter the spectral shape of fluorescence emitted from egg white proteins (Figure 2(Ca)), compared with crosslinked egg white protein MPs (Figure 2(Cb)).

Figure 2(Cc) shows the shift in the PQ/egg white protein MP signal to a lower wavelength position compared with the peak signal of PQ alone (Figure S2). Note that a peak on the shoulder around 380 nm could be observed in Figure 2(Cc), while a peak at 420 nm was observed in free PQ (Figure S2). This observation suggests that PQ molecules, located within the protein scaffold, may be sensitive to the environment provided by the egg white protein MPs [27], resulting in the observed hypsochromic shift. It also indicates that the PQ molecules were securely loaded within the 3D crosslinked structure of the egg white proteins. The emission peak of the GOx/PQ/egg white protein MPs was observed to undergo almost no shift (Figure 2(Cd)) when compared with the spectrum of PQ/egg white protein MPs, likely because of the dominance of fluorescence emission from GOx that overwhelms the fluorescence emission from PQ/egg white protein MPs.

Furthermore, we employed Fourier transform infrared spectroscopy (FTIR) to confirm the formation of GOx/PQ/egg white protein MPs, which provided information about functional groups and various bonds. Specifically, the IR spectra of egg white proteins prior to crosslinking with glutaraldehyde showed a broad peak at 3278 cm^−1^, corresponding to O-H stretching and N-H stretching, which are characteristic functional groups of proteins (Figure 2(Da)). Moreover, it showed a peak of C=O stretching (at around 1633 cm^−1^) and N-H bending (at around 1548 cm^−1^). After the crosslinking step using glutaraldehyde, the intensity of these two peaks decreased (at 1635 and 3346 cm^−1^) (Figure 2(Db)). Additionally, the C-H stretching (at around 2933–2959 cm^−1^), N-H stretching (at around 2874 cm^–1^), and C-H bending (at around 1380–1465 cm^–1^) were prominent when crosslinking with egg white proteins. This information suggested a successful crosslinking reaction between the egg white proteins and glutaraldehyde. The IR spectrum of PQ/egg white protein MPs showed a O-H stretching peak at around 3323 cm^−1^ (Figure 2(Dc)). Furthermore, these PQ/egg white protein MPs showed increasing intensity of the C-H stretching peak at around 2873–2958 cm^−1^. Moreover, the C-H bending peak (at around 1380–1465 cm^−1^) was still present with a slight increase in intensity. This suggests successful loading of PQ into the egg white protein MPs. After functionalizing the GOx (prepared in egg white proteins) in PQ/egg white protein MPs, the FTIR analysis revealed two distinct peaks: a O-H stretching peak and C=O stretching peaks at around 3280 and 1640 cm^−1^, respectively (Figure 2(Dd)). These two peaks were more prominent due to the addition of GOx along with additional egg white proteins. Therefore, the addition of GOx and egg white proteins enhanced the intensity of the O-H stretching and C=O stretching peaks, attributable to the functional groups present in GOx and egg white proteins. Additionally, this large addition obscured other peaks associated with those particular functional groups.

### 3.3. Electrochemical Characterizations

**Figure 3:**
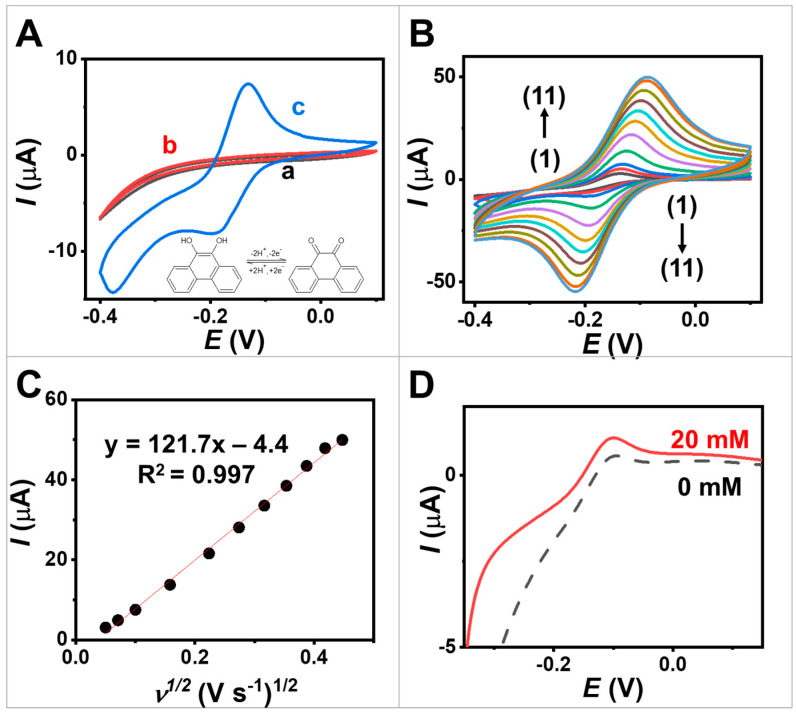
Electrochemical studies. (**A**) CVs obtained at scan rate 10 mV s^−1^ from (**a**,**black**) screen-printed CNT-modified electrode, (**b**,**red**) screen-printed electrode coated with egg white protein MPs, and (**c**,**blue**) screen-printed electrode coated with PQ-containing MPs. (**B**) CVs obtained from screen-printed electrode coated with PQ-containing MPs at different scan rates ranging from 2.5 to 200 mV s^−1^. The scan rates (1–11) used were 2.5, 5, 10, 25, 50, 75, 100, 125, 150, 175 and 200 mV s^−1^. (**C**) Plots of anodic peak current in function with square root of the scan rate obtained on a screen-printed electrode coated with PQ-containing MPs. (**D**) Linear sweep voltammetry (LSV) obtained from screen-printed electrode coated with GOx/PQ-based MPs at 0 and 20 mM glucose with a scan rate of 5 mV s^−1^.

To assess the electrochemical performance of the egg white protein MPs and PQ-containing MPs, CV was employed. Three distinct working electrodes were investigated: (a) a bare screen-printed electrode, (b) an electrode coated with egg white protein MPs, and (c) an electrode coated with PQ/egg white protein MPs. The CV curve in Figure 3A, obtained at a scan rate of 10 mV s^−1^, reveals the background current of the screen-printed electrode when evaluating in 0.1 M PBS at pH 7.0, serving as the supporting electrolyte (indicated by (a)). This indicates the stability of the screen-printed electrode within a potential window ranging from −0.40 to 0.10 V. The observed increase in cathodic current may be attributed to the oxygen reduction reaction occurring at a higher cathodic potential (initiating around −0.20 V).

The impact of loading PQ molecules in MPs on the electrochemical properties was investigated. The PQ is a redox mediator as it can undergo reversible oxidation and reduction reactions, facilitating electron transfer during electrochemical processes [22]. The inclusion of PQ in egg white protein MPs resulted in the emergence of distinctive redox peaks in the voltammogram, corresponding to the oxidation and reduction of PQ (represented by the blue line, curve c in Figure 3A), in contrast to the screen-printed electrode and egg white-protein-MP-modified electrodes (curves a and b). The oxidation and reductions peak were observed at −0.14 V and −0.20 V. This notable increase in peak currents could be attributed to the redox activity of PQ. The well-defined shapes of the redox peaks in Figure 3A, curve c, indicate the two-electron reduction and oxidation process of PQ at the electrode modified with the PQ-containing MPs [28]. Note that PQ molecules contain high aromaticity, leading to π–π stacking interactions between PQ and the screen-printed carbon nanomaterials, particularly at sp^2^ carbon in CNTs, ensuring a strong and effective interaction. This suggests the successful immobilization of PQ on egg white protein MPs and the redox performance of the modified electrode using the synthesized MPs.

Furthermore, we estimated the capacitance of the electrodes by considering the cyclic voltammogram (CVs). For more details, refer to Equation (S1), Equation (S2), and Supporting Note S1. This evaluation enables us to determine the amount of charge involved in the electrochemical reaction. The specific capacitance values of screen-printed CNT-modified electrode, egg white protein MPs, and PQ-containing MPs were 0.2, 0.2, and 1.5 mF cm^−2^, respectively. This result demonstrates that the attachment of PQ enhances the charge storage capabilities through successful reversible redox reactions on the electrode.

Figure 3B illustrates the CVs of screen-printed electrode coated with PQ-containing MPs, with a scan rate ranging from 2.5 to 200 mV s^−1^ in 0.1 M PBS pH 7.0. These plots reveal the presence of quasi-reversible PQ redox couples, and the anodic/cathodic peak current ratio (I_c_/I_a_ ratio) approaches unity, ranging from 1.0 to 1.1. The CV shapes remain well preserved, indicating that the screen-printed electrode coated with PQ-containing MPs exhibits good rate performance. Furthermore, as depicted in Figure 3C, the resulting anodic and cathodic peak currents show a proportional relationship to the square root of the scan rate. This behavior aligns with the Randles–Sevcik equation [29] and suggests that the processes occurring at the PQ/egg white-protein-MP-modified electrode are diffusion-controlled, consistent with previous studies conducted on a PQ-modified electrode [30].

Additionally, we studied the effect of scan rate on the surface capacitance by considering the area under the cyclic voltammogram, with PQ serving as the redox-active molecules attached to the electrode (Figure S3A,B). We observed a decrease in capacitance from 2.64 to 0.56 mF cm^−2^ when the scan rate increased from 2.5 to 200 mV s^−1^. Faster scan rates during CV resulted in a decrease in capacitance because of the limited time available for redox reactions to occur at the electrode surface.

Additionally, we measured CVs using the bare electrode, egg white protein MPs, and PQ-containing MPs in 0.1 M KCl, with and without an additional external redox. In the KCl electrolyte, the PQ-containing MPs exhibited a higher redox peak current compared with the bare electrode and egg white protein MPs. This difference is due to the presence of PQ as a redox mediator (see inset Figure S6A). When the solution contained an external redox probe, we observed a relatively smaller redox peak current when using the electrode modified with egg white protein MPs and PQ-containing MPs, compared with the bare electrode (Figure S6A). This decrease can be attributed to the insulating nature of egg white protein and the GOx enzyme, which restricts the accessibility of the external redox probe in reaching the electrode. Furthermore, we compared the active surface area of the pristine commercial conductive ink with a screen-printed CNT-modified electrode (see Figure S6B and Supporting Note S2). The redox peak of the screen-printed electrode was higher than that of the commercial conductive electrode. The active area of the screen-printed electrode was 0.12 cm^−2^, while that of the commercial electrode was 0.09 cm^−2^. This indicates that the modification can increase the active area by approximately 25% compared with the pristine electrode. Additionally, the EIS measurements showed that our ink composed of CNT exhibited a lower resistance value than the commercial ink (see Figure S7). This enhancement can be attributed to improved electron transfer.

To evaluate the bioelectrocatalytic characteristics of the GOx/PQ-based MPs, while minimizing the contribution of capacitive current resulting from coated materials and the large electroactive area of the CNT-based electrode, the LSV technique was performed at a low scan rate of 5 mV s^−1^. The LSV technique was used to vary the potential of the screen-printed electrode coated with GOx/PQ-based MPs linearly with time while measuring the resulting current. The voltammograms shown in Figure 3D (dashed line) display an anodic peak around 0.15 V, indicating the oxidation of PQ molecules in the absence of glucose. This voltammogram reflects the oxidation behavior of PQ. In the presence of glucose, the oxidation peak current increased (Figure 3D, solid lines). The anodic peak observed during LSV was higher compared with the absence of glucose, indicating the effective catalytic action of GOx, functionalized in the synthesized MPs. The presence of glucose led to an increased anodic peak in the LSV, enabling quantitative measurement of the glucose levels. Amperometry was further employed to demonstrate glucose biosensors using these GOx/PQ-based MPs (Section 3.4 Amperometry). This observation confirms the successful electrochemical behavior of a screen-printed electrode coated with GOx/PQ-based MPs upon the addition of glucose. This is a result of effective enzyme immobilization and PQ mediation within the microspheres.

**Figure 4:**
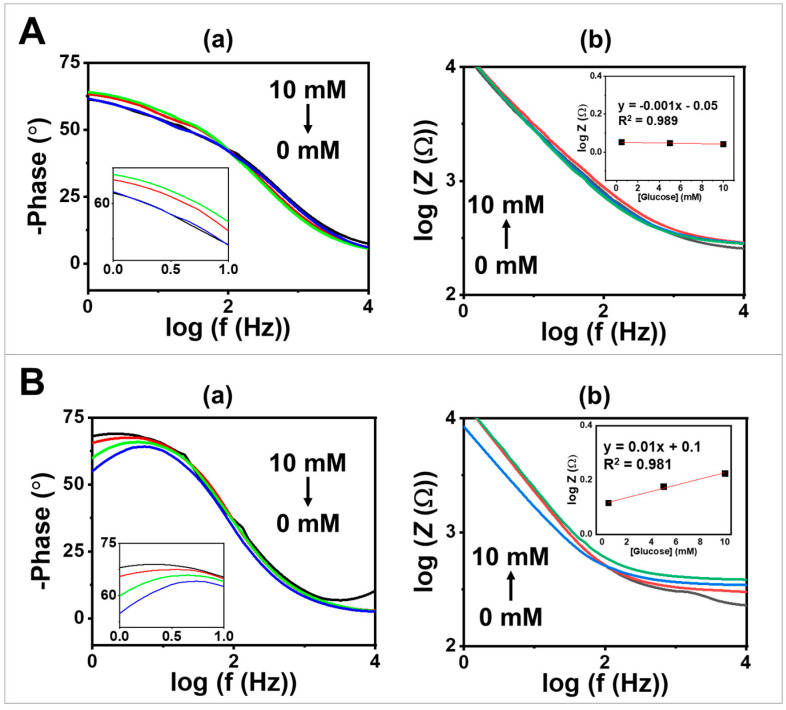
EIS studies of glucose sensor on (**A**) a screen-printed CNT-modified electrode compared with (**B**) screen-printed electrode coated with GOx/PQ/egg white protein MPs at glucose concentrations of 0, 0.5, 5, and 10 mM in 0.1 M PBS with a pH of 7.0, using a frequency range of 10^0^–10^4^ Hz, an amplitude of 5 mV, and 0.2 V DC; (**a**) phase angle vs. log f at frequency between 10^0^ and 10^4^ Hz. (**b**) Bode plots at frequency 10^0^–10^4^ Hz. The insets present a glucose calibration plot using log Z obtained at a frequency of 10^4^ Hz.

In addition to DC techniques, EIS is one of the techniques that has been widely used, particularly in chemical and biosensing applications [31]. In this study, EIS was conducted to evaluate the bioelectrochemical performance of the synthesized GOx-based MPs towards changes in glucose concentration. This evaluation was performed by comparing a bare electrode (Figure 4A) to a screen-printing electrode coated with GOx/PQ/egg white protein MPs (Figure 4B). Phase angle plots, as shown in Figure 4(Aa,Ba), were observed across a frequency range of 10^0^ to 10^4^ Hz and with varying glucose concentrations ranging from 0.5 to 10 mM. The phase angle values were found to be around 50–75° over a frequency range of 10^0^ to 5 × 10^1^ Hz, which is lower than the expected 90° for ideal capacitive behavior [32]. Figure 4(Ab) displays a Bode plot including the total impedance (log Z) as a function of the logarithm of the frequency depicting different glucose concentrations. The slope of the bare screen-printed electrode was determined to be −0.8 based on data at a frequency of 10^0^–10^2^ Hz. Additionally, the inset calibration graph in Figure 4(Ab) illustrates the relationship between log Z at a frequency of 10^4^ Hz and glucose concentration. The inset plot shows that the impedance signal obtained from the bare electrode does not depend on changes in glucose concentration due to the absence of glucose-sensitive particles.

When comparing the performance of a screen-printed CNT-modified electrode to an electrode modified with GOx/PQ/egg white protein MPs, the impedance signal increased with increasing glucose concentration from 0.5 to 10 mM. The slopes of the log Z vs. log f graph obtained for screen-printing electrodes coated with GOx/PQ-based MPs were determined to be −0.7 (over a range of 10^0^ to 10^2^ Hz) (Figure 4(Bb)), indicating pseudocapacitive behavior in the mid-frequency region. At high-frequency regions (over around 300 Hz), the slopes approached zero, indicating resistive behavior at those frequencies [32]. Furthermore, the calibration graph (shown in the inset in Figure 4(Bb)), which depicts the correlation between the logarithm of impedance (log *Z*) recorded at a high frequency of 10^4^ Hz and glucose concentration, exhibited an upward trend in the impedance signal as the glucose concentration increased. When using GOx/PQ/egg white protein MPs, the sensitivity for glucose detection (measured in mM^−1^ unit) was enhanced by approximately 43 times compared with the bare electrode. These results confirm the glucose-sensitive behavior of the synthesized GOx-based protein MPs. Additionally, these findings highlight the beneficial effects of electrode modification in improving the electrochemical performance of glucose sensing applications. Additionally, we compared the Bode plot generated from experimental data with the fit of the EIS data (Figure S4). The Randles circuit was applied to simulate the EIS data (Figure S5). The circuit consists of a resistor (*R*_el_) representing the electrolyte resistance, a constant phase element (*CPE*_dl_), or a capacitor (*C*) for the double layer for non-ideal behavior at the electrode–electrolyte interface, and a Warburg element (*W*_D_) describing the semi-infinite diffusion of the species in the electrolyte towards the electrode surface.

### 3.4. Amperometry

**Figure 5:**
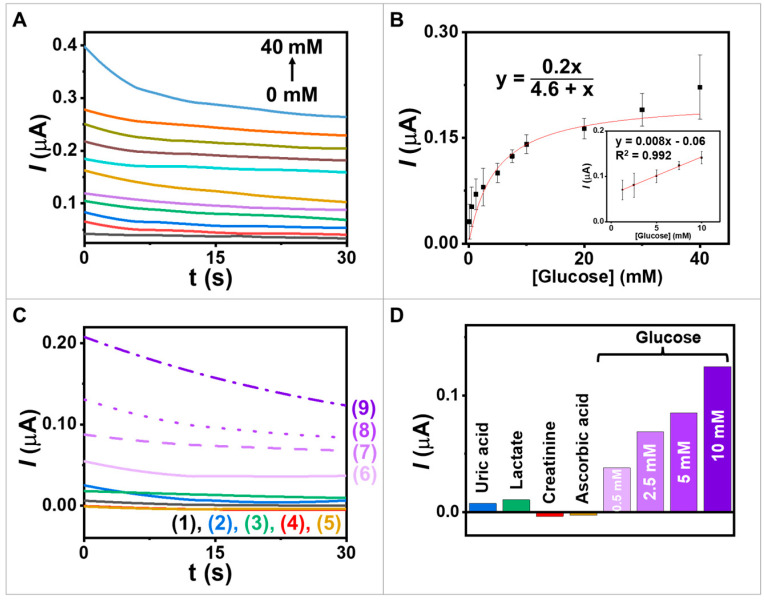
Study of electrochemical enzyme kinetics and selectivity. (**A**) Amperograms obtained from the screen-printed electrode coated with GOx/PQ/egg white protein MPs with an applied potential of 0.2 V vs. Ag/AgCl; glucose concentrations at 0, 0.125, 0.5, 1.25, 2.5, 5, 7.5, 10, 20, 30, and 40 mM. (**B**) The calibration plot between 0.125 and 40 mM and inset calibration in the range 1.25−10 mM. (**C**) Interference study in (1) blank and in the presence of (2) 2 mM uric acid; (3) 12.5 mM lactate; (4) 0.5 mM creatinine; (5) 100 mM ascorbic acid; (6–9) 0.5, 2.5, 5.0, and 10.0 mM glucose; potential step to 0.2 V (vs. Ag/AgCl). (**D**) The chart displays the responses towards glucose and other substances.

Investigating the electrochemical enzyme kinetics of redox-mediated GOx reactions is crucial as it helps determine the enzyme’s affinity for glucose and its maximum catalytic efficiency. In our study using a heterogeneous system, we employed an amperometric technique to examine the interaction between GOx and glucose (substrate) and to measure the rate of electron generation over time (Figure 5A). By incrementally adding glucose concentrations ranging from 0.125 to 40 mM while maintaining a potential of 0.20 V vs. Ag/AgCl, we correlated the current responses with the glucose concentration (Figure 5B). Moreover, the calibration in the inset of Figure 5B shows a linear range of glucose concentration from 0.125 to 10 mM with a sensitivity of 0.008 µA mM^−1^. The Michaelis–Menten constant ($K_{m}$) serves as an important indicator of enzyme-substrate kinetics. The results showed that $K_{m}$ was 4.6 mM, indicating that the immobilized GOx exhibited high enzymatic activity and that the proposed electrode had a strong affinity for glucose. The current responses exhibited the characteristic features of the Michaelis–Menten kinetic mechanism (Equation (1)). In enzymes that follow the Michaelis–Menten mechanism, increasing substrate concentrations in the initial phase led to a rapid increase in the current, followed by a gradual increase as the enzyme approaches its maximum activity. At high substrate concentrations, the enzyme becomes saturated, and the maximum achieved current reflects the enzyme–substrate complex being fully formed. Thus, the resulting curve represents the kinetic parameters defining the upper and lower boundaries of substrate concentration. Therefore, *K_m_* serves as an important parameter reflecting the enzyme–substrate affinity and influences the reaction velocity at various glucose concentrations.

(1) $$I=\frac{I_{max}[glucose]}{K_{m}+[glucose]}$$

where I is the steady-state current after the addition of glucose, $I_{max}$ is the maximum current obtained from saturated glucose concentrations, [glucose] is the glucose concentration, and $K_{m}$ is the Michaelis–Menten constant.

To validate our results and to obtain a deeper comprehension of the enzymatic properties, we compared the *K_m_* value calculated for our biosensor with values reported in the literature for similar enzyme–substrate systems on amperometric biosensors. For example, in the comparison of *K_m_* values, the encapsulation of GOx within spindle-like copper hydroxysulfate nanocrystals facilitated the precipitation Cu^2+^ ions and biomimetic mineralization of brochantite [33]. The *K_m_* of the free enzyme was compared with that of GOx@copper hydroxysulfate nanocrystals, yielding *K_m_* values of 19.01 mM and 14.39 mM, respectively. Another example, a CNT-chitosan-nanowire electrode was immobilized with GOx, resulting in tight immobilization within the matrix through adsorption [34]. The enzyme electrode exhibited a low *K_m_* value of 7.1 mM, which can be attributed to synergistic augmentation from CNT, chitosan, and the GOx matrix. The *K_m_* value of these papers were higher than that in our work, which obtained a weaker binding affinity between the enzyme and substrate, requiring higher substrate concentrations to reach half of the maximum reaction velocity. Additionally, a glucose sensor was developed using zinc oxide nanowires immobilized with GOx, which led to a *K_m_* value of 4.1 mM [35]. The observation of similar *K_m_* values with our work in different enzyme–substrate systems suggests comparable affinities between immobilized GOx and glucose. 

Investigating the selectivity of the new biosensor for glucose is crucial due to the presence of various physiologically significant interferences in real samples, such as a saliva sample that contains glucose, uric acid, lactate, ascorbic acid, and creatinine. To determine the biosensor’s selectivity, Figure 5C,D were used to assess the impact of different potential interferences on the response to 2 mM uric acid [36], 12.5 mM lactate [36], 0.5 mM creatinine [37], and 100 mM ascorbic acid [38]. These used concentrations were around five times as high as those informed in the literature. The selectivity test results indicate that the presence of coexisting compounds has little effect on the glucose response. For this experiment, the GOx/PQ/egg white protein MPs, based on a screen-printed electrode, could measure glucose using amperometry techniques at a low applied voltage in electrochemical analysis, resulting in reduced electrical input and power consumption [39]. Utilizing low applied voltages can help prevent electrode degradation and enhance electrode stability, as high voltages may induce side reactions [40]. Additionally, low voltages can enable better control over the desired reaction and enhance process selectivity, as certain reactions may exhibit different mechanisms or pathways at high voltages, leading to unwanted by products or decreased selectivity due to interference effect from substances, e.g., ascorbic acid and uric acid [41]. Furthermore, functionalizing enzymes in MP environments allows for achieving a high level of specificity [42]. When enzymes are immobilized or encapsulated within MPs, the enzymatic reactions occur in a controlled and confined environment. This microenvironment enhances the enzyme’s specificity by facilitating substrate recognition and catalytic activity. The MP environment provides protection to the enzymes from harsh conditions or interference from other molecules, thereby increasing their stability and preserving their specificity. Therefore, the use of MP environments offers significant advantages in attaining and maintaining high enzyme specificity across glucose biosensor applications.

### 3.5. Glucose Biosensors on Degradable Electrodes

**Figure 6:**
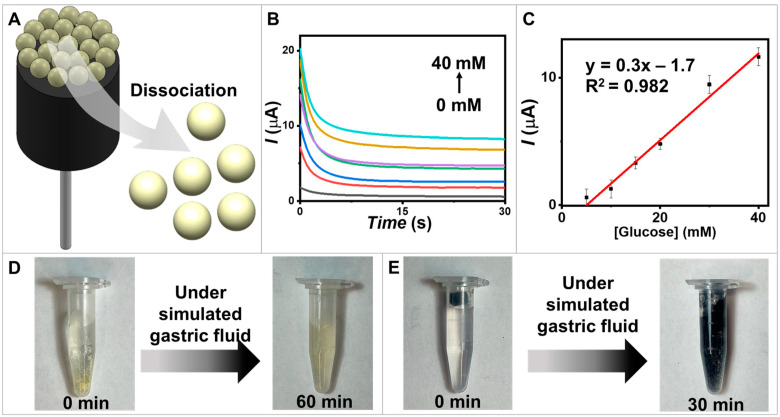
Degradable biosensors. (**A**) A schematic diagram illustrating GOx/PQ/egg white protein MPs based on degradable biosensors. (**B**) Amperograms of the degradable electrode coated with the GOx/PQ/egg white protein MPs were recorded for a duration of 30 s, using an applied potential of 0.6 V vs. Ag/AgCl. The recordings were taken for various glucose concentrations at 0, 5, 10, 20, 30, and 40 mM. (**C**) The calibration linear range plots at glucose concentrations 5–40 mM and error bars (*n* = 3). (**D**) The degradability of the GOx/PQ/egg white protein MPs in a simulated gastric environment, assessed at 0 and 60 min. (**E**) The degradability of the CNT/olive oil electrode coated with glucose-sensitive MPs and chitosan in a simulated gastric environment, assessed at 0 and 30 min.

Degradable electrodes are gaining interest due to their environmentally friendly and inexpensive nature [4]. They allow for the recycling of nanomaterials, approaching the concept of zero waste, and offer great exploration potential for the future. It is intriguing to further investigate materials that can support the development of decomposable electrodes. In this regard, we attempted to incorporate the use of food materials. Our degradable carbon electrode was constructed using CNT and edible olive oil to enhance biodegradability and ecological safety. Furthermore, the decomposable CNT/vegetable oil combination was modified with biodegradable functional MPs. In this study, our aim was to develop an electrode coupled with glucose-sensitive MPs for glucose detection. The materials used in all components were designed to dissociate when exposed to acidic conditions and agitation, which mimic environments found in the human stomach (Figure 6A).

The biosensing performance of the presented degradable biosensors was demonstrated through chronoamperometric measurements to monitor glucose levels with an applied potential of 0.6 V (Figure 6B). A linear relationship between the measured oxidation current and the glucose concentration was observed across the range of 0–40 mM (Figure 6C). To demonstrate the degradable capability of the electrode material, which refers to its ability to undergo controlled degradation or dissolution over time in a specific environment, we conducted experiments on the dissociation of GOx/PQ-based MPs and carbon electrodes. The dissociations of materials were observed at 60 min for GOx/PQ-based MPs (Figure 6D) and 30 min for the degradable carbon electrode (Figure 6E). Biosensors designed for degradation over time could experience dissociation in acidic conditions, such as the gastric environment, due to chemical alterations or structural degradation. Both the electrodes and protein materials are susceptible to degradation in this acidic and mechanically agitated environment. These demonstrations represent the potential of degradable electrodes to reduce environmental problems from undecomposed materials and the successful development of a biodegradable glucose biosensor.

## 4. Conclusions

We synthesized new food-based protein MPs and functional GOx/PQ/egg white protein MPs, using CaCO_3_ templates. This approach of utilizing food-based proteins to form homogeneous microspheres showed promising potential for glucose biosensor applications. The egg white protein MPs provided a biocompatible and supportive matrix for incorporating the enzyme and redox molecules. By incorporating PQ as a redox mediator, the biosensor enhanced the electrochemical response and sensitivity for glucose detection. We combined GOx with egg white protein MPs to create sensitive biorecognition layers, as demonstrated by impedance AC mode and amperometry DC mode. These functional protein MPs were integrated onto screen-printed CNT-modified electrodes and decomposable edible olive oil/carbon electrodes. This combination offered benefits, including a simple and cost-effective manufacturing method for the biosensor. Additionally, the integration of GOx/PQ-based MPs and olive oil-based carbon electrodes enabled degradation under simulated gastric conditions, emphasizing the environmentally friendly nature and “green” applications of our materials. For future directions, it would be interesting to address the remaining challenges associated with the use of enzymatic materials in complex situations. Additionally, it is important to investigate a large population of real matrices in biofluids. By developing cheap, biodegradable, biocompatible materials; improving enzyme immobilization techniques; and exploring practical applications, it is conceivable that such concepts can unlock new potential for these materials in various applications.

## Data Availability

The data are contained within the article and the Supplementary Materials.

## Supplementary Materials

The following supporting information can be downloaded at https://www.mdpi.com/article/10.3390/bios13080772/s1, the supplementary figures and sections in the research study focus on the characterization and electrochemical studies of the materials used in the glucose sensor. Figure S1. optical microscopic images of different materials. Figure S2. the fluorescence emission spectrum of PQ alone. Figure S3. the apparent capacitance profiles at various scan rates. Figure S4. demonstrates EIS studies of the glucose sensor. Figure S5. the equivalent circuit of EIS for the screen-printed electrode. Figure S6. electrochemical studies of the different electrodes. Figure S7. EIS studies of screen-printed CNT-modified and commercial conductive carbon electrodes. Supporting Note S1. The areal capacitance calculation. Supporting Note S2. The electroactive surface area of the electrode.
