# Supplementary material for: Glucose Oxidase/Egg White Protein Microparticles with a Redox Mediator for Glucose Biosensors on a Screen-Printed Electrode and a Decomposable Electrode
Source: Biosensors (Basel). 2023 Jul 29;13(8):772. doi: 10.3390/bios13080772 (PMC10452649; doi:10.3390/bios13080772)
Supplement: Supplementary file 1 [file biosensors-13-00772-s001.zip › supporting information-20230728.pdf]

Supporting Figures

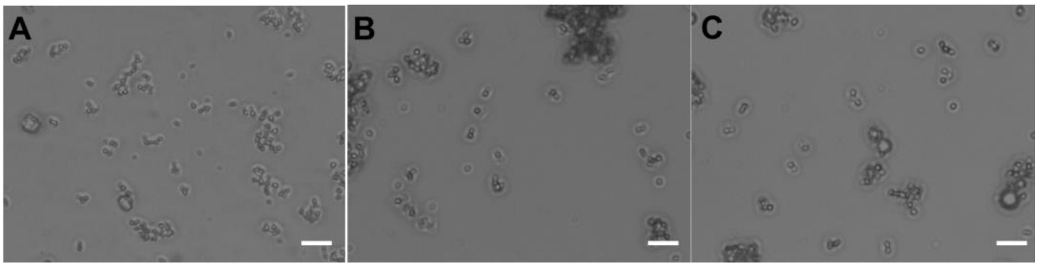

Figure S1. Characterization of materials. The Optical microscopic image of (**A**) $CaCO_3$ microparticles loaded with egg white proteins, (**B**) egg white protein MPs, and (**C**) PQ/egg white protein MPs. Scale bar: 10 μm.

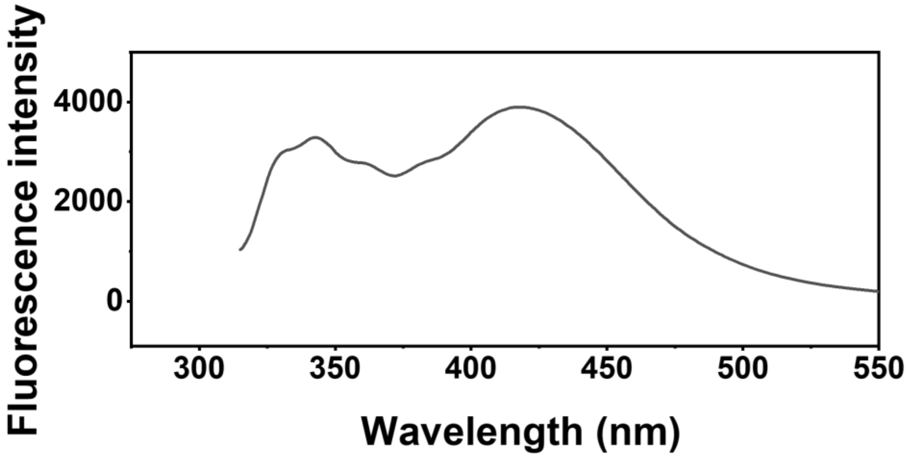

Figure S2. Fluorescence emission spectrum of PQ alone at emission between 315-550 nm and fixed excitation wavelength at 300 nm.

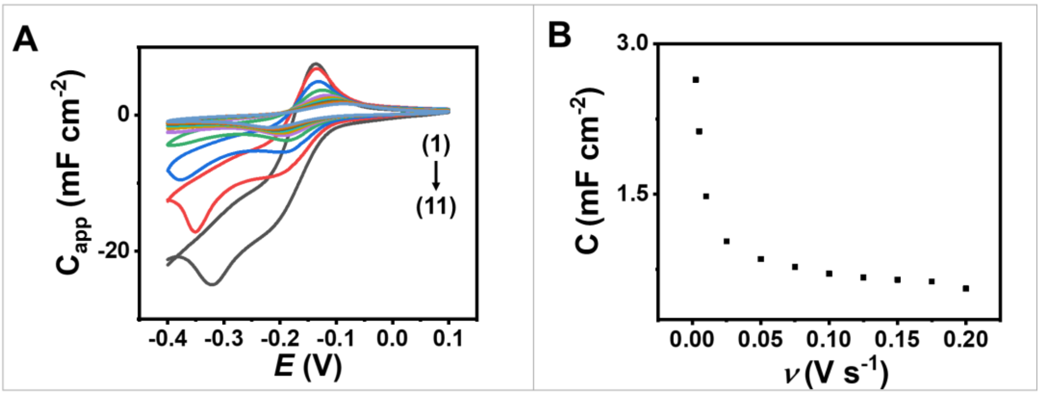

Figure S3. (**A**) Apparent capacitance profile in 0.1 M PBS pH 7.0 solution at different scan rates ranging from 2.5–200 mV s^{-1}. The scan rates (1–11) used were: 2.5, 5, 10, 25, 50, 75, 100, 125, 150, 175, and 200 mV s^{-1}. (**B**) Plot the capacitance of the screen-printed electrode coated with PQ-containing MPs at scan rates ranging from 2.5–200 mV s^{-1}.

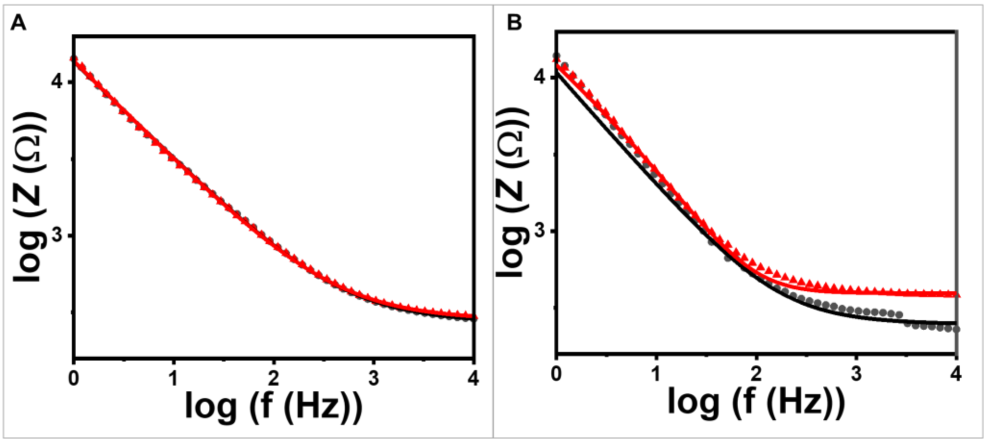

Figure S4. EIS studies of glucose sensor on (A) a screen-printed CNT-modified electrode compared with (B) screen-printed electrode coated with GOx/PQ/egg white protein MPs at glucose concentrations of 0 (black line) and 10 mM (red line) in 0.1 M PBS with a pH of 7.0, using a frequency range of 10^0–10^4 Hz, an amplitude of 5 mV, and 0.2 V DC; Bode plots at frequency 10^0–10^4 Hz. The real data was depicted in the symbol line, while the solid line represents the Rundles circuit diagram, which is a potential model for fitting the data.

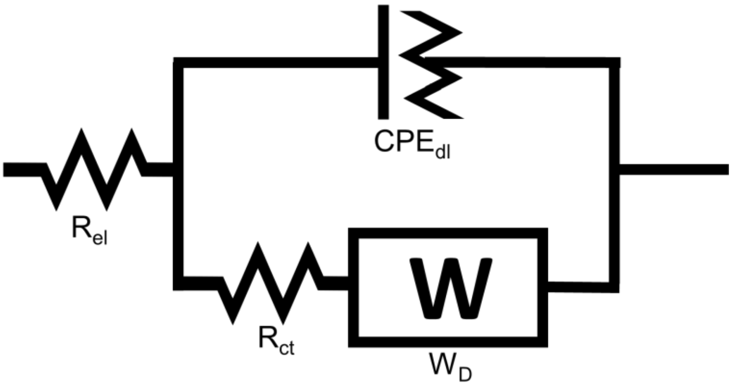

Figure S5. Equivalent circuit of EIS for screen-printed electrode coated with GOx/PQ/egg white protein MPs.

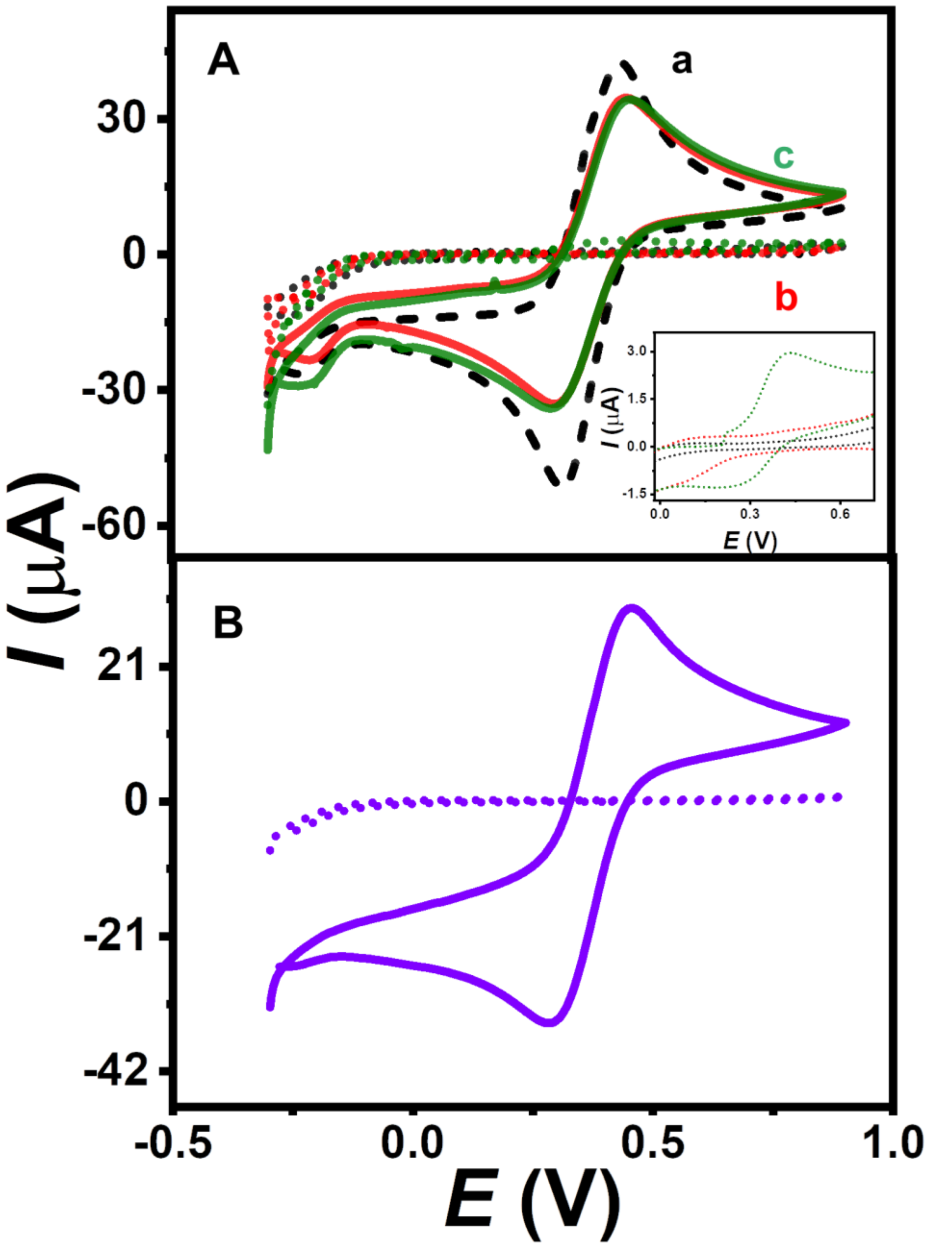

Figure S6. Electrochemical studies. (**A**) CVs obtained at a scan rate of 10 mV s^{-1} in 0.1 M KCl without 5 mM $K_3[Fe(CN)_6]$ (dot lines) and with 5 mM $K_3[Fe(CN)_6]$ (solid line) from (a, black dash line) screen-printed CNT-modified electrode, (b, red solid line) screen-printed electrode coated with egg white protein MPs, and (c, green solid line) screen-printed electrode coated with PQ-containing MPs. The inset presents the CVs of electrodes in 0.1 M KCl without 5 mM $K_3[Fe(CN)_6]$ (dot lines). (**B**) CVs obtained at scan rate of 10 mV s^{-1} from a commercial conductive carbon ink in 0.1 M KCl (dot lines) without 5 mM $K_3[Fe(CN)_6]$ and with 5 mM $K_3[Fe(CN)_6]$ (solid line).

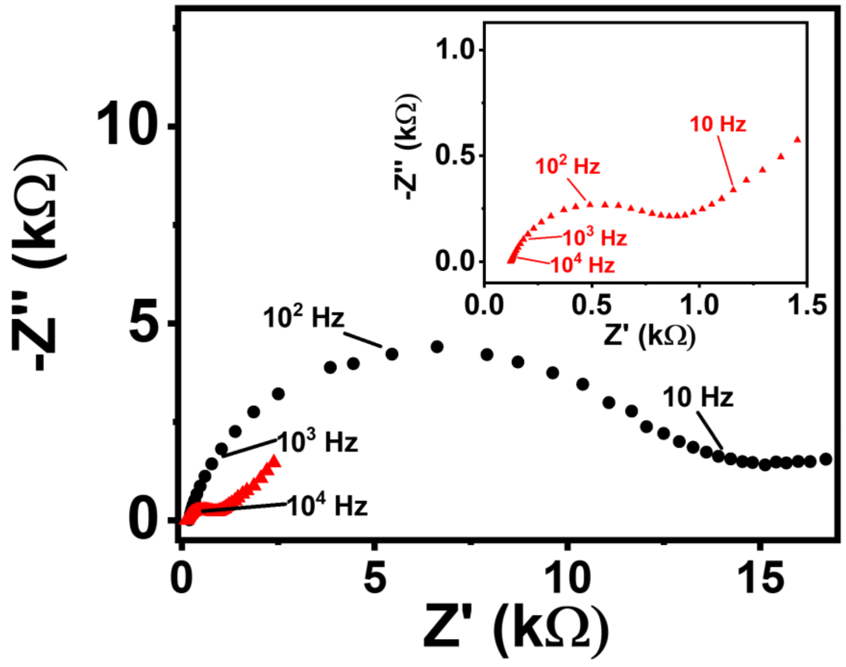

Figure S7. EIS studies of a screen-printed CNT-modified electrode (red line) compared with a commercial conductive carbon electrode (black line) in a mixing solution of 5 mM potassium hexacyanoferrate (III) in 0.1 M KCl and 5 mM potassium hexacyanoferrate (II) trihydrate in 0.1 M KCl, using a frequency range of 10^{-1}–10^{5} Hz, an amplitude of 10 mV, and 0.3 V DC. The real data was depicted in the symbol line, while the solid line represents the Rundles circuit diagram, which is a potential model for fitting the data.

Supporting Notes

S1. The areal capacitance calculation

The areal capacitance of an electrode was estimated according to CVs data and calculated by Supporting Equations S1 and S2.

$$C = \frac{1}{2 \times (V_2 - V_1) \times v} \int_{V_1}^{V_2} |I(V)|\, dV \qquad \text{(Equation S1)}$$

$$C_{areal} = \frac{C}{A} \qquad \text{(Equation S2)}$$

For the calculation of specific capacitance (F cm^{-2}) from cyclic voltammetry measurements, where *I(V)* is the instantaneous current in each potential (A), V_2 and V_1 are the upper and lower potential of the chosen potential window (V), v is the scan rate (V s^{-1}), and A is the geometrical area of the electrode (cm^2). Additionally, the resulting specific capacitance is further divided by the area of the working electrode (0.15 cm^{-2}).

S2. The electroactive surface area of the electrode

The surface of a screen-printed CNT-modified electrode can be studied by estimating the electroactive surface area of the electrode used. Applying the Randles-Sevcik equation (Equation S3), the obtained different CVs indicate the difference in the surface area of a screen-printed CNT-modified electrode and commercial conductive carbon ink.

$$I_p = 2.69 \times 10^5 n^{3/2} A D_0^{1/2} C_0 v^{1/2} \qquad \text{(Equation S3)}$$

Where I_p is peak current (A), n indicates the number of electrons transferred (n = 1 for $K_3[Fe(CN)_6]$), A is the surface area of the electrode (cm^2), D_0 is the diffusion coefficient (D_0 = 7.6 × 10^{-6} cm^2 s^{-1} for $K_3[Fe(CN)_6]$), C_0 is the concentration of electroactive species (mol cm^{-3}), and v is the scan rate (V s^{-1}).
